# Cyanide Toxicity of Freshly Prepared Smoothies and Juices Frequently Consumed

**DOI:** 10.9734/ejnfs/2018/44004

**Published:** 2018-09-03

**Authors:** A. Baker, M. C. Garner, K. W. Kimberley, D. B. Sims, J. H. Stordock, R. P. Taggart, D. J. Walton

**Affiliations:** 1 Department of Life Sciences, University of Nevada, Las Vegas, NV 89154, USA.; 2 Department of Physical Sciences, College of Southern Nevada, N. Las Vegas, NV 89030, USA.; 3 Department of Biological Sciences, College of Southern Nevada, Las Vegas, NV 89146, USA.

**Keywords:** Cyanide, smoothie, juice, amygdalin, linamarin, flax seed, almonds

## Abstract

**Aims:**

This study was conducted to detect the presence of cyanide in popular fruit and vegetable smoothies and juices marketed as raw and natural.

**Study Design:**

Eleven (11) popular varieties of drinks were analyzed for total cyanide (_T_CN). Drinks contained raw vegetables and fruits, flax seeds, whole apples with seeds, raw almond milk, and pasteurized almond milk as ingredients.

**Place and Study duration:**

Samples were collected from health food eateries located within Las Vegas, Nevada (USA) during the summer of 2017.

**Methodology:**

Fifty milliliters (mL) of a homogenized smoothie and juice drink and 1 gram of flax seeds were subjected to the above-referenced methods for sample preparation per USEPA Methods 9012B (digestion) followed by USEPA method 9014 (colorimetry).

**Results:**

The highest _T_CN was detected in drinks containing raw flax seed followed by unpasteurized raw almond milk, then fresh whole apple juice. No _T_CN was observed in drinks that contained none of the above mentioned items (e.g. flax seed, raw almond milk) or those utilizing pasteurized ingredients.

**Conclusion:**

This study observed that _T_CN is present in smoothies and juices containing raw flax seeds, fresh whole apples, and/or unpasteurized almond milk. Concentrations were detected as high as 341 μg L^−1^ in commercially available smoothies containing vegetables, raw flax seeds, almond milk and fruits. Smoothies with vegetables, fruits, unpasteurized almond milk, and no flax seeds contained 41 ug L^−1^_T_CN, while similar smoothies with pasteurized almond milk contained negligible to 9.6 ug L^−1^ CN^-^. Unpasteurized almond milk and raw flax seeds were the major sources of _T_CN in drinks. With the increased demand for raw and natural foods, there is a potential sublethal exposure of _T_CN by consumers.

## 1. INTRODUCTION

The dangers of CN^-^ to living organisms is well documented; yet, it is still used in various applications including polymer synthesis, metallurgy, extraction of precious metals, and commercial herbicides [1,2,3]. It is also found in many plant foods, fruits, and kernels (i.e., seeds) in the form of cyanogenic glycosides (α-glycosides or β-glycosides), which are secondary metabolites consisting of α-hydroxynitrile and a sugar derivative [4,5]. While anions are known to play a vital role in the environmental and biological processes of living organisms, CN^-^ derived from cyanogenic glycosides are toxic and can be damaging to organisms when consumed in large enough quantities [4,6,7]. Lee et al., [5] and others have shown that anionic species (cyanogenic glycosides as CN^-^) are greater in size with diverse shapes containing higher hydration energies within a wide-ranging scale of hydrophobicity, ultimately impacting living organisms [4].

It is known that humans are exposed to low levels of CN^-^ daily from vehicle exhaust, water sources, foods, and smoking cigarettes [4]. The United States Environmental Protection Agency (USEPA) has set a Maximum Contaminant Level (MCL) for CN^-^ (0.2 mg L^−1^) in drinking water due to evidence showing nerve and thyroid damage [8]. It is estimated that the general nonsmoking, nonurban population in the United States is exposed to 3.8 μg CN^-^ per day from atmospheric sources and 0.4–0.7μg CN^-^ found in well-water, assuming consumption of ~2 liters per day [9].

Exposure also comes from naturally occurring cyanogenic glycosides or cyanoglycosides found in foods [10,11]. Studies have identified at least 55 different cyanogenic glycosides in over 2,650 plant species, many of them used in everyday foods [9,10]. Cyanide in such food items is known to be a metabolic product of bacteria, fungi, and algae. Many plant species of beans, fruits (e.g. apple and cherry seeds, almonds, cashew), and flax seed (*Linum usitatissimum*) contain various forms and concentrations of CN^-^ [11,12,13]. Due to the plethora of CN^-^ containing plants, the World Health Organization (WHO) has been unable to estimate the total amount of CN^-^ consumed on an average day per person [14].

**Fig. 1a.**
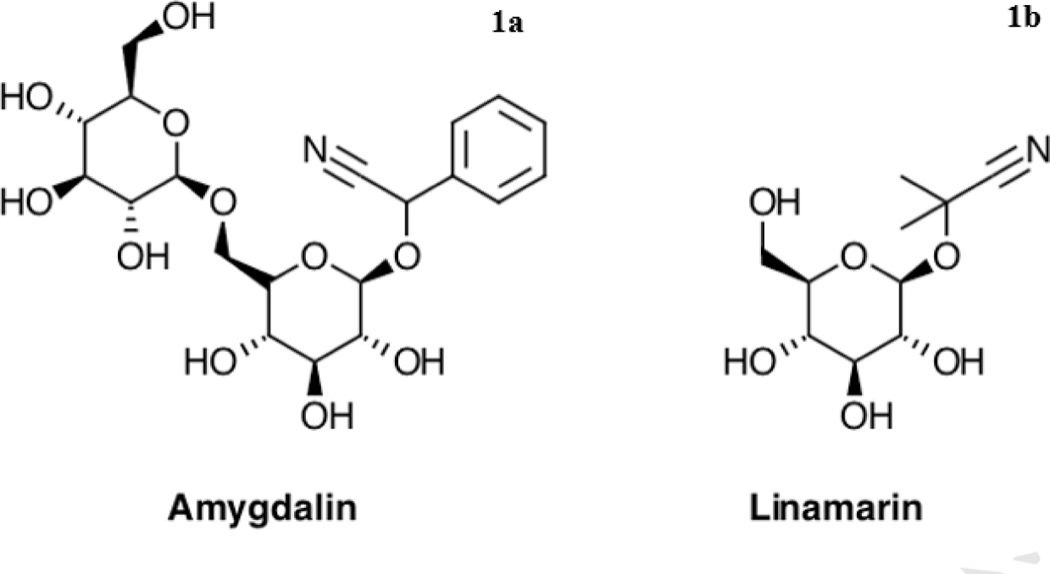
Cyanide form (“Amygdalin”) found in apple seeds and almonds; 1b: Cyanide form (“Linamarin”) found in flax seeds

Raw and natural foods such as seeds and nuts have become a large part of human nutrition with the “eat raw and natural” push over the past decade with a reported 40% of adults consuming raw seeds and nuts daily [4,15]. Most forms of CN^-^ in health foods originate from amygdalin (Fig. 1a) contained in apple seeds and almonds or, linamarin (Fig. 1b) contained in flax seeds [4]. Many of these items are used in the health food industry (e.g., fresh smoothies and juices) as a selling point for improving one’s fitness, vigor, and strength as they are a good source of omega-3 fatty acids, lignans, and fiber [4]. Other promotional advantages of these items put into smoothies and juices include weight loss, and improved skin and hair, resulting in an increase of more than 80% in the past 5 years [15]. Moreover, most of these diets have a low protein component which increases the risk of chronic, sublethal CN^-^ related effects according to Bolarinwa et al. [4] and others [16]. With this increased demand for these health drinks and additives such as flax seeds added for their omega-3 fatty acids, antioxidants, and fibre, there is a potential for sublethal exposure to CN^-^, leading to a variety of chronic health related consequences [17].

### 1.1 Cyanide Toxicity

When exposed to cyanogenic glycosides, an organism’s breakdown of CN^-^ is dependent on the presence and amount of hydrolytic enzymes [18,19,20]. Other authors have shown that when there are not enough hydrolytic enzymes to break down CN^-^, a lethal (“oral”) dose of CN^-^, or acute toxicity, can be as much as 0.54 mg CN^-^ kg^−1^ of body weight [9]. Studies have illustrated that CN^-^ ranging between 0.5 and 3.5 mg kg^−1^ of body weight can also lead to acute poisoning [4]. Its (CN^-^) affect works by binding to the trivalent iron found in cytochrome c of the oxidative phosphorylation pathway, preventing the cell from utilizing oxygen, resulting in cellular hypoxia and decreased adenosine triphosphate (ATP) production [9,16]. Cyanide forces cells into anaerobic metabolism to produce ATP, resulting in a buildup of lactic acid, leading to an acid-base imbalance causing metabolic acidosis [21,22]. In addition to binding with cytochrome c, CN^-^ inhibits free radical scavengers such as catalase and peroxidase, producing further oxidative stress, phosphatase preventing ATP production, and succinic dehydrogenase reducing the production of CoQ10 that is needed for producing cellular energy and removing free radicals [21,23].

The primary targets for CN^-^ toxicity are the cardiovascular, respiratory, and central nervous systems [24]. The central nervous system, especially the brain, is the most significantly affected [21]. Studies of populations that rely primarily on cassava for dietary starches have shown that chronic consumption can lead to sublethal CN^-^ exposure. This exposure can result in severe neurologic effects such as hyperreflexia or spastic paraparesis of the extremities known as Konzo, spastic dysarthria, visual and hearing difficulties, cerebellar signs, and memory impairment [17,21,23,25,26,27].

Soto-Blanco et al. [20] explained that CN^-^ is ubiquitous in the environment and when exposed to unborn goat fetus there are embryo-toxic and teratogenic effects, however, the LD_50_ for CN^-^ exposure was established in animal studies and is insufficient to provide a full assessment of risk to human fetuses. Moreover, due to complications related to CN^-^ poisoning and the lack of data concerning the teratogenicity of CN^-^, it is not currently possible to state that absence or presence of maternal CN^-^ exposure excludes any adverse effects to the development of a human fetus or during breast feeding. However, animal studies clearly showed that fetal effects did exist between mother and fetus due to the transfer of CN^-^ during the pregnancy [20]. Finally, Soto-Blanco and Gorniak [18] also showed that there is a transfer of CN^-^ from mother to goats (kids) through suckling (breast feeding) of milk.

The popularity of raw almonds, flax seeds and other foods deemed as healthy have been rising with little to no oversight by regulatory agencies. Cyanide toxicity in foods is not a new issue; it has been documented that people have died due to consuming raw almonds in excess [12,28]. The purpose of the present study was to examine CN^-^ levels within health food drinks (i.e., fresh smoothies and juices) being promoted as healthy.

This study evaluated the potential exposure of CN^-^ by consumers drinking freshly prepared smoothie and juice drinks purchased at health food eateries in the Las Vegas, Nevada (USA) market. Drinks (“Samples”) were collected from popular eateries and analyzed for total CN^-^ utilizing USEPA approved methods. The primary goals of this study were to 1) evaluate smoothies for CN^-^ content by analyzing samples within 1 hour of preparation; 2) discus CN^-^ source in beverage components; and 3) discus exposure to an average person consuming such beverages.

## 2. MATERIALS AND METHODS

Raw and fresh drinks (i.e. smoothie and juices) are made with whole fruits (i.e. apples), almond milk, protein alternatives, seeds, and other items deemed healthy alternatives. Drinks were purchased locally from a variety of health food eateries within Las Vegas, Nevada between May and July 2017. A total of eleven (11) popular varieties of drinks were analyzed for total CN^-^ (_T_CN) concentration (Table 1a) over this period. Drinks contained either raw flax seeds, whole apples with seeds, raw almond milks or, pasteurized almond milk as specified in ingredients. Since flax seeds are an additive for their reported health benefits, brown and yellow flax seeds (smoothie additive) were collected from smoothie eateries and analyzed for _T_CN (Table 1b). The intent behind testing flax seed separately was to compare _T_CN concentrations detected in drinks with and without flax seeds. Apple seeds were not analyzed separately as they are part of the whole apples and are not additive but rather, part of the apple when juiced. Additionally, two beverages (E1 and F1) were analyzed for _T_CN that are sold as healthy alternatives to high sugar drinks for children and contain pasteurized apple and apricot juice. These two juice drinks were analyzed to evaluate the effectiveness of pasteurization on natural contaminants.

Drinks and flax seeds were analyzed per USEPA Methods 9012B (digestion) and 9014; distillation was followed by colorimetry. Fifty milliliters (mL) of each smoothie and juice drink (homogenized) along with 1 gram of flax seeds were subjected to the above referenced methods for analysis. The method (9012B and 9014) was modified for this study with an additional 90 minutes for digestion and distillation plus another 15 minutes for cool down. This method extracts hydrogen CN^-^ (i.e., hydrocyanic acid) as _T_CN from samples with H_2_SO_4_ followed by absorption of _T_CN in a gas scrubber using a 0.25N sodium hydroxide solution. A buffer of sodium dihydrogen phosphate (1M NaH_2_PO_4_-H_2_O) was added for stabilization followed by the additions of chloramine-T (C_7_H_7_C_l_NNaO_2_S) and pyridine-barbituric (C_5_H_5_N•C_4_H_4_N_2_O_3_) acid to generate color for analysis of CN^-^ via a Beckman Coulter UV colorimetry instrument at 578 nm wavelength. Methods utilized in this study were verified for the use of plant material and compared to other methods by Gleadow et al. [29] and by Ketterer and Keusgen [30].

Quality control was performed with two separate sources of solid potassium CN^-^ (KCN) purchased from two different USEPA approved vendors. Initial and continuing calibration verifications (Alfa Aesar, lot # 10193459) were performed with acceptable windows of 90–110%. Additionally, a laboratory control sample (LCS) was analyzed (Sigma-Aldrich, lot # MKBZ8253V) with acceptable windows of 85–115%. Calibration ranged from 0 to 600 μg L^−1^ with a method detection limit (MDL) established at 2 μg L^−1^ for this study per USEPA method 9014. All samples were analyzed in duplicate and digested followed by distillation per the method. Duplicate sample data were expressed as the relative percent difference (RPD) between duplicates with acceptable USEPA windows of ±20% [8].

## 3. RESULTS

Results for 11 popular smoothies and juices and two flax seed additives commonly used in these products are presented in Tables 2 and 3. In order of highest to lowest, _T_CN was detected in drinks containing flax seed followed by drinks with unpasteurized raw almond milk, and then fresh whole apple juice. No detectable _T_CN was observed in drinks that contained none of the previously mentioned items (e.g. flax seed, raw almond milk) or those that used pasteurized almond milk.

Data shows a wide range of _T_CN concentrations across drinks and within a single eatery where drinks were purchased on different days of the week. Freshly made smoothies (ID: B, D, E, F) viewed as providing a healthy alternative contained non-detectable (ND) CN^-^ to as much as 341 μg L^−1^_T_CN (ID: A2). Smoothies prepared with flax seeds contained the highest concentrations while smoothies with no flax seeds contained measurable CN^-^, though significantly lower than those with flax seeds. Freshly juiced apples (E2) contained low concentrations of CN^-^ (2.7 μg L^−1^), while prepackaged juices (IDs: E1and F1) and those made with no flax seeds (B2, D1, D2) contained no detectable _T_CN.

Samples purchased from the same eatery were inconsistent between drink mixtures with smoothie A1a and A1b contained 272 and 134 μg kg^−1^, respectively. Both smoothies (A1a and A1b) were collected from the same eatery, one-week apart. This difference was likely the result of inconsistency in drink preparation by eateries staff. Samples with flax seeds contained the highest _T_CN, while drinks with no flax seeds contained the lowest; flax seed contains linamarin, a natural source of CN^-^ [4]. Samples having low levels of _T_CN and no flax seed was made from freshly juiced apples with seeds, a source recognized to contain CN^-^ in the form of amygdalin [4]. Both sources of CN^-^ are liberated from apple seeds during the juicing process, leaving detectable levels in drinks.

Flax seed is added to smoothies as either whole or ground seeds for their reported health benefits, blended for homogeneousness to release nutrients, and consumed. Sample G1 (brown flax) contained 60 mg kg^−1^, while sample G2 (yellow flax) contained 51 mg kg^−1^ of _T_CN. It was observed that smoothies prepared with fresh whole apples, and no flax seeds also contain CN^-^ (i.e. A1c 9.6 μg L^−1^ and E2 2.7 μg L^−1^_T_CN), though considerably lower than those with flax seeds as illustrated in A1a, A1b, A2, A3, and B1 with 272, 124, 341, 158, and 205 μg L^−1^, respectively. Moreover, smoothies with added flax seeds were 1 order of magnitude higher in _T_CN than smoothies containing no flax seeds.

## 4. DISCUSSION

It has been observed that consumption of foods containing CN^-^ (e.g. raw almonds) causes intercellular hypoxia when it binds to mitochondrial cytochrome c oxidase a3 after consumption [13]. Eleven (11) freshly prepared smoothies and juices along with 2 flax seed additives were evaluated for total _T_CN concentrations to determine the risk of this food deemed a healthy alternative to processed foods. Eight (8) of the 11 drinks contained various levels of CN^-^ranging from 2.7 to 341 μg kg^−1^_T_CN. Flax seeds, a popular health food additive to these drinks, contained between 51 and 60 mg kg^−1^_T_CN. Studies have shown that ingredients such as apple and flax seeds contain amygdalin and linamarin, two of the most common forms of cyanogenic glycosides found in raw foods [4,15].

Flax seeds are marketed as a super-food promoting fitness, vigor, strength, hair growth and other benefits [4]. Studies suggest that natural foods including flax seeds not be consumed during pregnancy or, breastfeeding, due to possible effects on the unborn baby [31]. These findings show that drinks containing flax seeds had the highest levels of CN^-^ ranging from 134 to 341 μg kg^−1^_T_CN. Drinks with no flax seeds ranged between ND and 41 μg kg^−1^_T_CN. Levels of _T_CN in this study suggest that such consumption during pregnancy or breastfeeding may pose a sublethal exposure to the unborn child.

Linamarin, a cyanogenic glucoside in flax seeds is also in the leaves and roots of plants such as cassava and lima beans [4,15]. Authors describe linamarin as a glucoside of acetone cyanohydrin that when consumed raw, can cause adverse effects on the body due to its _T_CN content [9]. Other researchers have reported that upon exposure to endogenous β-glycosidase enzyme in the human gut, linamarin and its methylated relative lotaustralin can decompose to HCN, creating a possible avenue to sublethal exposure [27,32,33].

Although most parts of a plant can contain cyanoglycosides, studies have shown it to be concentrated in the seeds [27]. Apple seeds contain amygdalin (cyanogenic glycosides) and when juiced release low levels of _T_CN. Studies have shown that the seeds of apples contain low levels of _T_CN, this study showed that drinks with apple seeds and no flax seed contained between ND to 9.6 ug L^-1^. When a drink contained flax seeds CN^-^ levels were significantly higher than those made with just wholes apple. It is known that when a drink is blended, the juicing process liberates _T_CN ^-^ as a result of hydrolysis upon contact with water and enzymes and, from the vigorous blending and aeration [12,16]. However, the hydrolysis has limited impact with higher concentrations of _T_CN sources such as raw flax seeds leaving detectable levels. It is also known that microorganisms in the gastrointestinal system also produce the enzymes β-glycosidase and hydroxynitrile lyase that convert glycosides into HCN; another mechanism creating a pathway to exposure [16,27,32].

It is important to point out that linamarin is only partially metabolized (~25%) to HCN with the remaining unchanged linamarin excreted by the kidneys [34]. Animal researchers evaluated dogs and rats by feeding a linamarin (e.g., cassava) diet that led to the development of diabetes mellitus [32,34,35). They also determined that cyanide from linamarin can cause hypertrophy of the adrenal gland, leading to decreased function and hypoadrenocorticism [27,36]. Cyanide can also impact the adrenal gland function, resulting in weight gain and the risk of developing diabetes mellitus [27]. Therefore, diets focused on weight loss can have the opposite effect, causing the consumer to gain weight as a result of CN^-^ intake.

The metabolic product thiocyanate, while less toxic to the body than _T_CN, is not completely innocuous. Thiocyanate competes with iodine uptake and utilization by the thyroid gland, leading to the formation of goiters and hypothyroidism [37]. Cliff et al [37] also explain that hypothyroidism leads to weight gain, furthering the effects of _T_CN on the development of diabetes mellitus. Therefore, people with thyroid disease may experience further problems with their thyroid gland.

A noteworthy concern with linamarin is when there are dietary deficiencies in sulfur-containing proteins. The liver enzyme rhodenese, or thiosulfur transferase, uses sulfur to convert _T_CN to the less toxic thiocyanate that is excreted by the kidneys [16]. Approximately 80% of absorbed _T_CN metabolized to thiocyanate is excreted from the body within 24 hours according to Carlson [38] with the rate of detoxification by the body estimated to be 1μg kg^−1^ body weight/min [9]. If sulfur is deficient due to diet, the rate of detoxification slows, exacerbating the effects of _T_CN [16,34,38].

Since CN^-^ is metabolized by the body, the median lethal dose (LD_50_) is time dependent. This means that the LD_50_ is greater for chronic exposure verses acute exposure [21]. Slight effects have been noted with _T_CN levels ranging between 20 and 40 μg kg^−1^ body weight, whereas levels of 50–60 μg kg^−1^ body weight can be tolerated without immediate or later effects between 20 and 60 minutes after exposure [9]. Based on this information, an oral minimum risk level of 0.05 mg kg^−1^ CN^-^ of body weight/day has been established for an intermediate duration of exposure with no long-term exposure limits established [21]. Thus, an average 70 kg adult can consume up to 3.5 mg _T_CN over the course of a day without developing overt clinical signs of cyanide toxicosis. The highest concentration of _T_CN in this study contained 341 μg L^−1^ (sample A2) in a standard size of 650 mL - equaling 221 μg (0.221 mg _T_CN per serving) per standard size. An adult would have to consume 16 regular sized smoothies in less than two hours to be lethal [39]. This study is not suggesting that a person can consume a lethal dose through smoothies in two hours but rather, sublethal amounts over an extended period of time.

Another important point to make is when smoothies containing freshly juiced vegetables, fruits and raw seeds (i.e., flax) are consumed by children as young as 3 years of age, they may have a greater risk for a sublethal effect. The average weight of a 3-year-old is 14 kg [37], consuming multiple smoothies equaling 0.221 mg _T_CN per serving would be enough to cause clinical signs of acute _T_CN toxicity (~3 regular sized smoothies). Although the _T_CN content in a single smoothie (0.221 mg _T_CN per serving) is below the detoxification level of 1 μg kg^−1^ body weight/min for producing overt clinical signs, researchers have shown that chronic low-level exposure (sublethal) can cause histologic effects leading to clinical problems over the course of time [9,16,36] A single drink will not cause an acute effect on the consumer however, people on a healthy-green diet, those with compromised hepatic systems or, an underdeveloped hepatic system, may experience long-term impacts from the consumption of these types of drinks.

## 5. CONCLUSION

The current study has observed that _T_CN is present in smoothies and juices containing raw flax seeds, fresh whole apples, and/or unpasteurized almond milk. Potentially any fruit or vegetable containing cyanogenic glycosides, linamarin and amygdalin, may be contributing to _T_CN content in health food drinks such as smoothies. Cyanide from linamarin has been linked to a variety of health issues such as diabetes mellitus, neurological deficits, sensory or memory impairments, and weight gain through damage to the adrenal gland function. Moreover, thiocyanate, a metabolic by-product of CN^**-**^, has been tied to goiter growth and hypothyroidism. The presence of CN^**-**^ in these drinks do not pose an acute threat of poisoning; however, this study suggests that a diet consisting of regular raw flax seeds, fresh whole apples, and/or unpasteurized almond milk, smoothie intake may result in chronic sublethal exposure to _T_CN. The average adult can mitigate CN- toxins consumed in their daily diets. Women who may become pregnant, currently pregnant and people with developing or, compromised immune systems should monitor or restrict their intake of drinks containing raw flax seeds and almonds or unpasteurized almond milk. Finally, additional research is required to fully understand the possible health effects that exist in unprocessed fresh foods.

## Figures and Tables

**Table 1a.** Smoothie and juice ingredients per drink

Drink type	ID	Ingredients

Smoothie	A1	Pasteurized almond milk, cocoa powder, dark chocolate chips, coffee beads, avocado, spinach, kale, collard greens, raw flax seed, raisins, whole red/green apple, vanilla, agave nectar, ice
Smoothie	A2	Cocoa powder, dark chocolate chips, banana, roasted peanuts, avocado, kale, spinach, raw flax seed, zucchini, chia, honey, vanilla, agave nectar, ice
Smoothie	A3	Green grapes, whole green apple, pineapple, orange, lime, wheat grass, kale, spinach, collard greens, ginger, raw flax seed, agave nectar, ice
Smoothie	B1	Strawberries, blueberries, cranberry, multi-vitamin, raw ground flax seed, whole grain oats, whey protein, Splenda, ice
Smoothie	B2	Blueberries, mango, banana, roasted almonds, protein, ice
Smoothie	C	Unpasteurized almond milk, spinach, banana, vanilla, syntha-6, soy protein, yogurt, ice
Smoothie	D1	Cucumber, fresh apples, spinach, grapes, Greek yogurt, roasted pumpkin seeds, lemon juice, ice
Smoothie	D2	Whole apples, pineapple, kale, spinach, chia seed, ice
Juice	E1	Packaged, pasteurized apple juice for babies
Juice	E2	Freshly juiced whole apples
Juice	F1	Packaged, pasteurized apricot juice

**Table 1b.** Raw flax seeds

Seed	ID	Type

Flax seed, whole	G1	Brown
Flax seed, whole	G2	Yellow

**Table 2.** CN^-^ concentration of juices and smoothies commonly consumed

Drink type	Product / type	ID	_T_CN μg L^−1^ ± RPD

Smoothie	Pasteurized almond milk, cocoa powder, dark chocolate chips, coffee beads, avocado, spinach, kale, collard greens, raw flax seed, raisins, whole red apple, vanilla, agave nectar, ice	A1a	272 ± 17
Smoothie	Pasteurized almond milk, cocoa powder, dark chocolate chips, coffee beads, avocado, spinach, kale, collard greens, raw flax seed, raisins, whole red apple, vanilla, agave nectar, ice	A1b	134 ± 0.1
Smoothie	Pasteurized almond milk, cocoa powder, dark chocolate chips, coffee beads, avocado, spinach, kale, collard greens, raisins, whole red apple, vanilla, agave nectar, ice	A1c	9.6 ± 2.4
Smoothie	Cocoa powder, dark chocolate chips, banana, roasted peanuts, avocado, kale, spinach, raw flax seed, zucchini, chia, honey, vanilla, agave nectar, ice	A2	341 ± 8.1
Smoothie	Green grapes, whole green apple, pineapple, orange, lime, wheat grass, kale, spinach, collard greens, ginger, raw flax seed, agave nectar, ice	A3	158 ± 0.5
Smoothie	Strawberries, blueberries, cranberry, multi-vitamin, raw ground flax seed, whole grain oats, whey protein, Splenda, ice	B1	205 ± 6.0
Smoothie	Blueberries, mango, banana, roasted almonds, protein, ice	B2	ND
Smoothie	Unpasteurized almond milk, spinach, banana, vanilla, syntha-6, soy protein, yogurt, ice	C	41 ± 6.5
Smoothie	Cucumber, fresh apples, spinach, grapes, Greek yogurt, roasted pumpkin seeds, lemon juice, ice	D1	ND
Smoothie	Whole apples, pineapple, kale, spinach, chia seed, ice	D2	ND
Juice	Packaged, pasteurized apple juice for babies	E1	ND
Juice	Freshly juiced whole apples	E2	2.7 ± 0.1
Juice	Packaged, pasteurized apricot juice	F1	ND

^ (A1b) purchased from same location as A1a, one week apart to evaluate consistency in formula. RPD is relative percent difference between duplicates samples. ND: non-detectable, _T_CN is total cyanide

**Table 3.** CN^-^ concentration in flax seeds used as additives to juices and smoothies

Seed	Product	Type	ID	_T_CN mg kg^−1^ ± RPD

Flax seed	Whole seed	Brown flax seeD	G1	60 ± 1.9
Flax seed	Whole seed	Yellow flax seed	G2	51 ± 4.6

Note: RPD is the relative percent difference between duplicates samples, _T_CN is total cyanide
